# Evaluation of the MDM-score system for screening mitochondrial diabetes mellitus in newly diagnosed diabetes patients: a multi-center cohort study in China

**DOI:** 10.3389/fendo.2024.1511101

**Published:** 2024-12-19

**Authors:** Fuhui Ma, Jing Zhao, Yan Chen, Yunzhi Luo, Yuxuan Du, Xia Li, Tao Xu, Zhiguang Zhou, Kaixin Zhou, Yanying Guo

**Affiliations:** ^1^ Graduate School, Xinjiang Medical University, Urumqi, Xinjiang, China; ^2^ Clinical Laboratory Center, Xi’an People’s Hospital Xi’an Fourth Hospital, Affiliated People’s Hospital of Northwest University, Xi’an, Shanxi, China; ^3^ National Clinical Research Center for Metabolic Diseases, Key Laboratory of Diabetes Immunology (Central South University), Ministry of Education and Department of Metabolism and Endocrinology, The Second Xiangya Hospital of Central South University, Changsha, Hunan, China; ^4^ Department of Endocrinology and Metabolism, People’s Hospital of Xinjiang Uygur Autonomous Region, Xinjiang Clinical Research Center for Diabetes, Xinjiang Key Laboratory of Cardiovascular Homeostasis and Regeneration Research, Urumqi, Xinjiang, China; ^5^ Guangzhou National Laboratory, Guangzhou, Guangdong, China; ^6^ School of Public Health, Guangzhou Medical University, Guangzhou, Guangdong, China

**Keywords:** mitochondrial diabetes mellitus, type 2 diabetes, MDM-score, screening, genetic testing, m.3243A>G, heteroplasmy

## Abstract

**Objective:**

To evaluate the performance of MDM-score system in screening for mitochondrial diabetes mellitus (MDM) with m.3243A>G mutation in newly diagnosed diabetes.

**Methods:**

From 2015 to 2017, we recruited 5130 newly diagnosed diabetes patients distributed in 46 hospitals in China. Their DNA samples were subjected to targeted sequencing of 37 genes, including the mitochondrial m.3243A>G mutation. Based on this cohort, we analyzed the clinical characteristics of MDM and type 2 diabetes (T2DM), and evaluated the overall efficacy of the MDM-score through ROC curve analysis.

**Results:**

MDM patients were diagnosed at a younger age (*P* =0.002) than T2DM patients. They also had a higher proportion of females, lower body mass index, lower height, lower weight, lower systolic blood pressure, and lower fasting C-peptide (*P* < 0.05). Among 48 MDM patients, the m.3243A>G heteroplasmy level was higher in MDM score ≥ 3 than in MDM score < 3 (*P* = 0.0281). There were 23 cases with MDM-score ≥ 3 in clinical T2DM, with an AUC of 0.612 (95% CI: 0.540-0.683, *P <*0.001) on ROC curve analysis, yielding sensitivity of 47.9%, specificity of 74.4%, positive predictive value of 1.9%, and negative predictive value of 99.3%. This suggests that almost half of MDM patients can be identified by the MDM score system.

**Conclusions:**

The MDM-score is effective for screening MDM in newly diagnosed clinical T2DM, and some metrics may help to improve its performance in the future, thereby assisting clinicians in identifying suitable patients for genetic testing, and preventing misdiagnosis and mismanagement of MDM patients.

## 1 Introduction

Mitochondrial diabetes mellitus (MDM) is a special type of monogenic diabetes caused by mutations in mitochondrial DNA (mDNA) or nuclear DNA (nDNA) that affect the mitochondrial respiratory chain with impaired oxidative phosphorylation and reduced ATP production, resulting in decreased insulin secretion by glucose-stimulated β cells (1–4). Among the mutations in MDM patients, m.3243A>G is the most common pathogenic mutation with a prevalence of nearly 85% (5, 6). It is estimated that this variant is carried by 0.2-2% of diabetes patients, with potentially higher prevalence among Asians compared to Europeans (7–10). Some patients with the m.3243A>G mutation present with severe early symptoms of mitochondrial myopathy, encephalopathy, lactic acidosis, and stroke, collectively known as MELAS syndrome (11). Due to the rapid progression of the disease, most patients with MDM develop complications earlier than those with type 2 diabetes mellitus (T2DM), including cardiovascular, renal, and ocular diseases (12–15). MDM patients typically manifests between the ages of 35 and 40 (16). Early clinical screening can help patients receive personalized treatment, improve prognosis, and guide genetic counseling. However, because of the low prevalence of MDM and the high costs associated with genetic testing, it is not reasonable to screen all diabetes patients. Consequently, the clinical selection of patients prioritized for genetic testing is primarily based on phenotype screening.

The phenotypic variability of MDM makes it likely to share features of both type 1 and type 2 diabetes, often leading to misdiagnosis of MDM (6, 16, 17). This variability is influenced by factors such as the level of heteroplasmy (the presence of both normal and mutated mitochondrial DNA) and its distribution across different tissues (18, 19). Clinical screening protocols for MDM are usually based on typical clinical phenotypes, including maternally inherited, the need for insulin therapy due to progressive pancreas β cell dysfunction, neurosensory deafness, and elevated serum lactate levels. However, not all patients with MDM present with every clinical phenotype simultaneously, and clinicians may overlook patients with atypical presentations. Therefore, it is crucial for clinicians to select the effective screening methods for the early detection of MDM in diabetic patients.

There is no consensus on the clinical screening criteria for MDM. Based on the clinical features of 1064 hospitalized Chinese patients with MDM, a 5-point MDM score screening tool was developed recently (20). With a cutoff of MDM-score ≥ 3, the screening tool achieved a sensitivity of 100% in hospitalized patients with clinical T2DM. However, its specificity may be limited due to the small sample size and the fact that most hospitalized patients had longer disease duration and more typical symptoms, the applicability of MDM-score to early stage MDM patients remains unclear.

Here, we used a large cohort of patients with newly diagnosed diabetes recruited from 46 hospitals across China. After excluding those with established MODY diagnosis or glutamic acid decarboxylase antibody (GADA) positivity, we investigated whether MDM-score could effectively identify candidates for MDM genetic testing, with the aim of exploring broader application scenarios for MDM- score.

## 2 Materials and methods

### 2.1 Participants

The study utilized data and samples from 46 tertiary care hospitals in 24 provincial administration areas in seven geographically diverse regions of China conducted between April 2015 and October 2017, as described previously (21, 22). All participants were ≥ 15 years, met the 1999 WHO criteria for the diagnosis of diabetes with a disease duration less than 1 year, were negative GADA and from outpatient clinic in the Department of Endocrinology of the 46 participating hospitals. Of the 17,114 participants who met above criteria, 5130 patients with early-onset diabetes aged ≤ 45 years were included in the analysis. The study was approved by the Ethics Committee of the Second Xiangya Hospital, Central South University in China (No. 2014032). Ethical approval of the study protocol was obtained from each participating hospital in accordance with the Declaration of Helsinki, and written informed consent was obtained from all participants. The examination included a series of anthropometric indicators, personal medical history, and biochemical tests. Their DNA samples were subjected to targeted next-Generation Sequencing (tNGS).

### 2.2 MDM-score screening criteria

The MDM-score consisted of the following 5 parameters for which a total score was calculated: (1) age at diagnosis of diabetes ≤ 45 years (1 point); (2) have a family history of diabetes and/or abnormal hearing ability on their mother’s side (1 point); (3) BMI < 24.0 kg/m^2^ (1 point); (4) abnormal hearing ability according to physical examination (1 point); and (5) impaired beta−cell function, there was endogenous insulin secretion, but < 11 μU/ml, or on insulin treatment (1 point). A total score of ≥ 3 was used as the screening cutoff value.

### 2.3 Genetic diagnosis

To detect nucleotide substitutions, a multiplex PCR panel was designed to amplify the coding regions ± 50bp of 37 genes (including the m.3243A>G) known to cause monogenic diabetes (**Supplementary Table S1**). The multiplex library was constructed using a two-step PCR method. The design of the custom assay, library preparation, sequencing, and data analysis were conducted as previously described to an average depth of 2000X. The heteroplasmy level of the mitochondrial variant m.3243A>G was calculated by counting the percentage of G3243 reads within all the valid sequencing calls at this site. Patients with a heteroplasmy level > 1% were defined as positive cases.

### 2.4 Statistics analysis

Demographic and clinical variables were reported as median (Q1-Q3) or mean ± standard deviation for continuous variables and number (%) for categorical variables. We used Student’s t-test to compare continuous variables if normal distributions were not rejected, and Mann-Whitney U test if normal distributions were rejected. The categorical variables between two groups were compared using Chi-square tests or Fisher’s test. All statistical analyses were performed using R, version 4.2.1 (R Programming). *P* values < 0.05 were considered statistically significant, and *P* values < 0.01 were considered highly significant.

The confusion matrix consisting of true positives (TP), true negatives (TN), false positives (FP), and false negatives (FN), which was established to calculate sensitivity, specificity, positive predictive value (PPV), and negative predictive value (NPV). Formulas were as follows: Sensitivity=TP/(TP+FN); Specificity= TN/(TN+FP); PPV = TP/(TP +FP); NPV = TN/(TN + FN). We measured the overall performance of the MDM-score using the area under the curve (AUC) of the receiver operating characteristic curve (ROC). All these analyses were performed using the reportROC package and ggplot2 package.

## 3 Results

### 3.1 Participants characteristics

**Figure 1:**
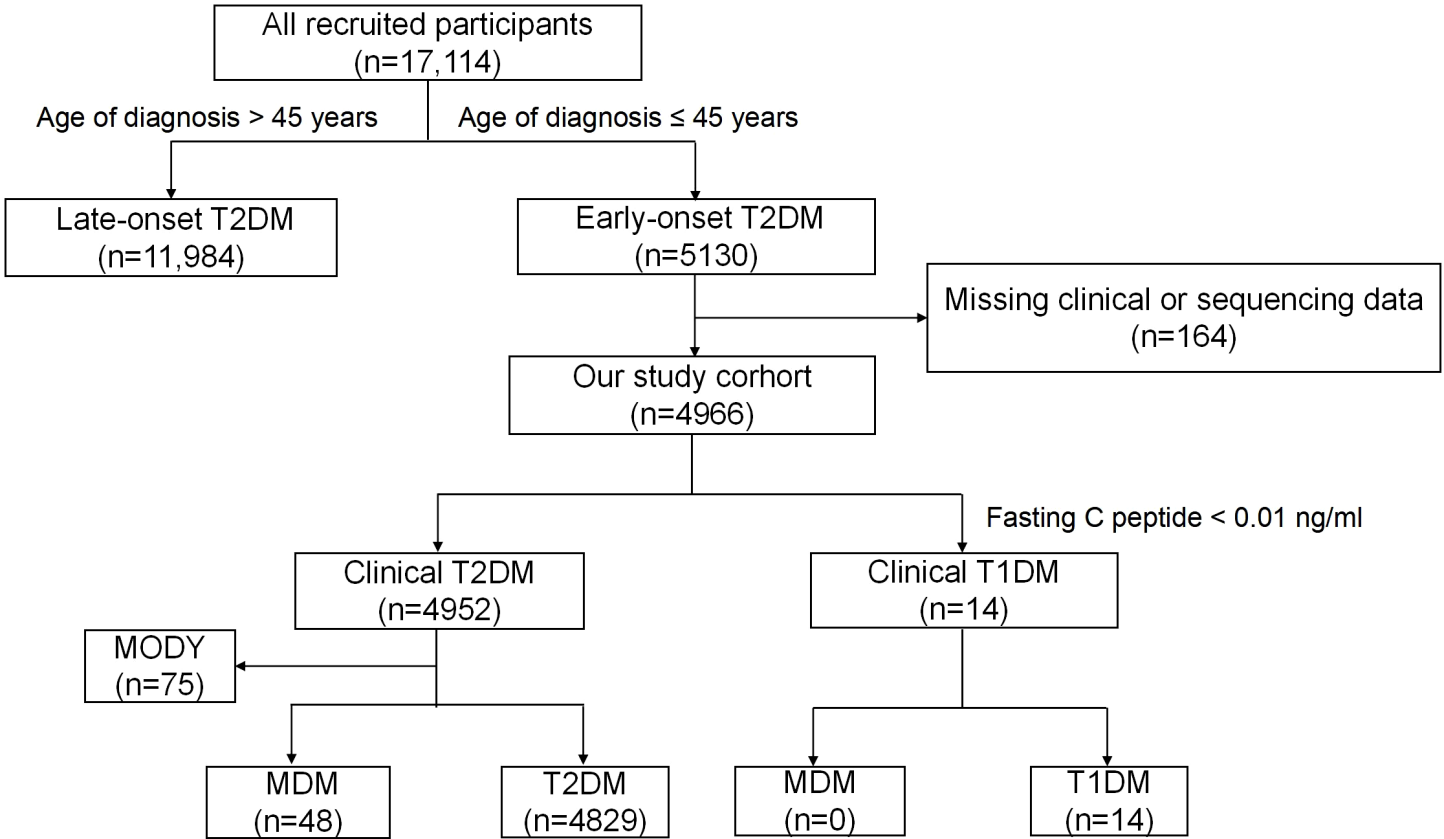
Flow chart of the study participants. MDM,Mitochondrial diabetes mellitus; T1DM,Type 1 diabetes mellitus; T2DM, type 2 diabetes mellitus; MODY, maturity-onset diabetes of the young; GADA, glutamic acid decarboxylase antibody.

**Figure 1** illustrates the progression and categorization of participants throughout the study. A total of 164 participants were excluded due to incomplete information. The study included 4966 eligible participants. Among them, 4952 were diagnosed with phenotypic T2DM, referred to as clinical T2DM, while 14 were diagnosed with phenotypic type 1 diabetes (T1DM), referred to as clinical T1DM. In the clinical T2DM cohort, genetic testing identified 75 cases (1.51%) of MODY and 48 cases (0.97%) of m.3243A>G MDM. Ultimately, 4829 participants received a definitive diagnosis of T2DM.

Patients were diagnosed with clinical T1DM if they met one of the following criteria: (1) severe insulin deficiency, defined as fasting serum C-peptide < 0.01 ng/mL; or (2) persistence of positive T1DM-related autoantibodies. All patients in this study tested negative for GADA and exhibited no clinical features of pancreatitis. Thus, 14 cases were classified as clinical T1DM due to fasting C-peptide levels below 0.01 ng/mL.

### 3.2 Characteristics between MDM and T2DM patients in clinical T2DM

**Table 1** shows the baseline characteristics of the two groups patients with MDM and confirmed T2DM. MDM patients were diagnosed at a younger age (31.5 *vs*. 36.4 years, *P* =0.002). They also had a higher proportion of females (43.8% *vs*. 27.4%, *P* = 0.017), a lower BMI (20.99 *vs*. 25.46 kg/m², *P* < 0.001), a lower height (162.30 *vs*. 168.27 cm, *P* < 0.001), a lower weight (55.94 *vs*. 72.54 kg, *P* < 0.001), a lower systolic blood pressure (117.39 *vs*. 124.03 mmHg, *P* = 0.002), and a lower fasting C-peptide (0.40 *vs*. 0.54 ng/mL, *P* = 0.026). While the MDM group showed a slightly greater prevalence of insulin treatment, family history of diabetes, and history of DKA than the T2DM group, these differences were not statistically significant (*P* > 0.05).

**Table 1:** Characteristics between MDM and T2DM patients in clinical T2DM.

Characteristics	MDM	T2DM	*P* Value
N	48	4829	
Female, n (%)	21 (43.8)	1321 (27.4)	0.017
Age of diagnosis (year)	31.5 (26.0-39.1)	36.4 (29.8-41.3)	0.002
Anthropometric factors			
Body mass index(kg/m^2^)	20.99 ± 4.14	25.46 ± 3.99	<0.001
Height (cm)	162.30 ± 8.80	168.27 ± 8.11	<0.001
Weight (kg)	55.94 ± 15.00	72.54 ± 14.35	<0.001
Systolic blood pressure (mmHg)	117.39 ± 11.74	124.03 ± 14.58	0.002
Diastolic blood pressure (mmHg)	77.17 ± 9.87	80.23 ± 10.79	0.056
Biochemical data			
HbA1c (%)	10.03 ± 2.96	9.70 ± 2.72	0.404
Fasting plasma glucose (mmol/L)	7.50 (6.80-10.23)	8.37 (6.59-11.70)	0.340
2 h Postprandial plasma glucose (mmol/L)	14.26 (10.24-19.42)	14.80 (11.19-18.95)	0.862
Fasting C-peptid (ng/mL)	0.40 (0.27-0.62)	0.54 (0.33-0.80)	0.026
2 h Postprandial C-peptide (ng/mL)	1.11 (0.67-1.78)	1.29 (0.75-2.04)	0.292
Triglyceride (mmol/L)	1.77(1.25-2.59)	1.87 (1.22-3.08)	0.280
Total Cholesterol (mmol/L)	4.87 ± 1.29	4.84 ± 1.39	0.899
High density lipoprotein (mmol/L)	1.07 (0.97-1.30)	1.05 (0.90-1.26)	0.116
Low density lipoprotein (mmol/L)	3.00 ± 0.88	2.85 ± 0.98	0.312
Questionnaire data			
Diet treatment, n (%)	19 (39.6)	2097 (43.4)	0.593
Physical activity treatment, n (%)	15 (31.3)	1804 (37.4)	0.384
Insulin treatment, n (%)	6 (12.5)	483 (10.0)	0.740
Metformin treatment, n (%)	12 (25.0)	1755 (36.3)	0.104
Sulphonyl treatment, n (%)	3 (6.3)	492 (10.2)	0.510
Acarbose treatment, n (%)	5 (10.4)	683 (14.1)	0.460
GLP-1 treatment, n (%)	1 (2.1)	108 (2.2)	1.000
Family history of diabetes, n (%)	18 (37.5)	1672 (34.6)	0.677
Diabetic ketoacidosis history, n (%)	8 (16.7)	527 (10.9)	0.204

Data were presented as number (%) for categorical variables, median (Q1-Q3) or mean ± standard deviation for continuous variables. MDM, Mitochondrial diabetes mellitus; T2DM, type 2 diabetes mellitus; HbA1c, hemoglobin A1c.

### 3.3 The proportion of MDM cases increased with MDM-score in clinical T2DM

**Figure 2:**
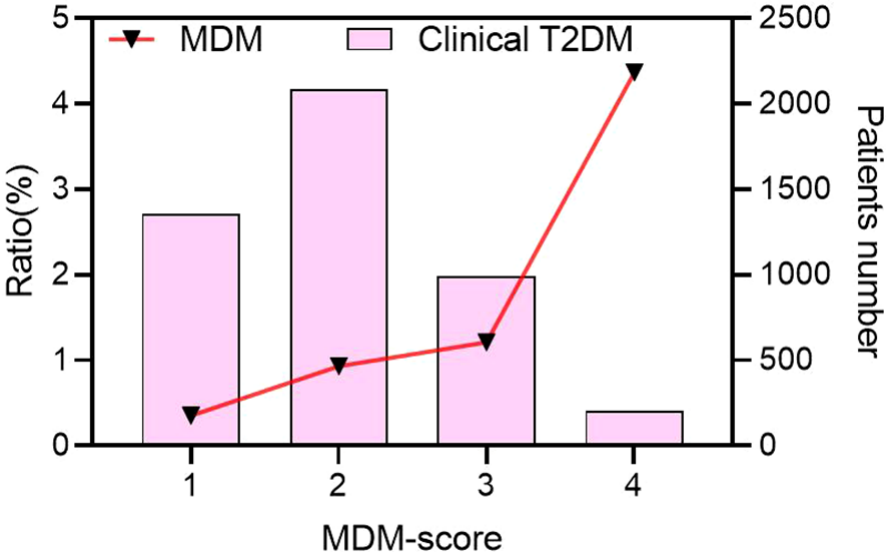
The proportion of MDM cases increased with MDM-score in clinical T2DM. MDM, Mitochondrial diabetes mellitus; T2DM, type 2 diabetes mellitus.

Among 48 MDM patients with the m.3243A>G mutation, 39.1% had a family history of diabetes, 82.6% had a BMI below 24.0 kg/m², and 32.6% were receiving insulin treatment. All patients denied experiencing hearing loss and did not undergo further electronic hearing testing. As a result, none of the patients had an MDM score of 5. The proportion of MDM cases increased steadily with higher MDM scores (**Figure 2**). There were 206 patients with scores of 4, including 9 cases with MDM (4.37%); 1152 patients with scores of 3, including 14 MDM (1.21%); 2,160 patients with scores of 2, including 20 MDM (0.93%); and 1,434 patients with scores of 1, including 5 MDM (0.35%) (**Supplementary Figure S1)**.

### 3.4 Characteristics and heteroplasmy level between MDM-score ≥ 3 and MDM-score 3 with MDM patients

**Figure 3:**
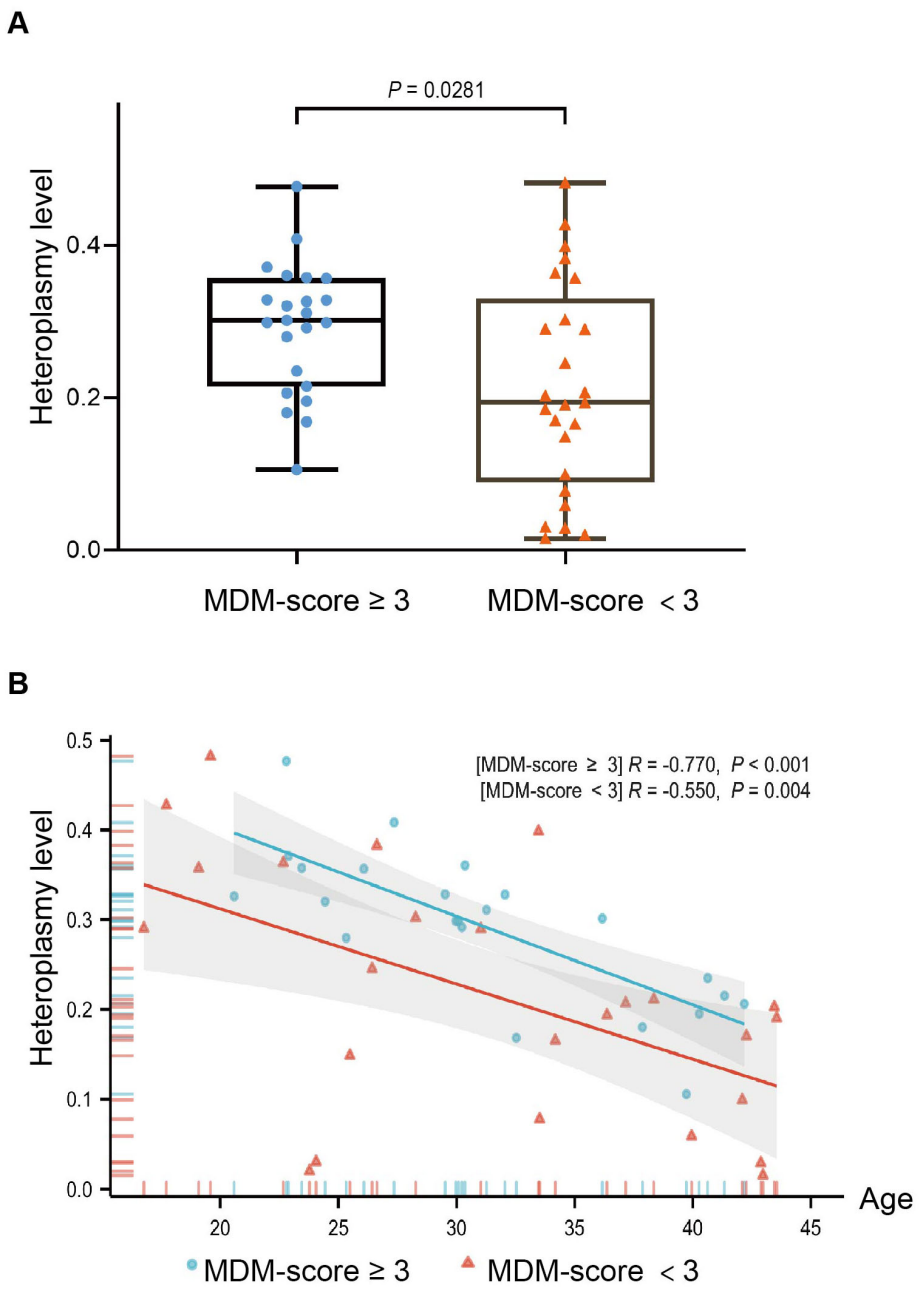
The distribution of the m.3243A>G heteroplasmy level with MDM-score in 48 MDM patients **(A)** Heteroplasmy level of the MDM-score ≥ 3 group was higher than the MDM score < 3 group (*P* = 0.0281); **(B)** Correlation scatterplot of two groups between heteroplasmy level and age at diagnosed diabetes(*P*<0.001; *P* = 0.004).

**Table 2** shows comparison of clinical characteristics between 23 patients with an MDM score ≥ 3 and 25 patients with an MDM score < 3. In addition to significant differences in BMI, insulin treatment, and family history of diabetes, we observed a notable difference in weight (50.60 kg *vs*. 59.97 kg, *P* = 0.031), suggesting that body weight may be a potential indicator for distinguishing MDM from type 2 diabetes. **Figure 3** illustrates the m.3243A>G heteroplasmy of blood in both groups. The heteroplasmy level was significantly higher in the MDM score ≥ 3 group compared to the MDM score < 3 group (*P* = 0.0281). Both groups exhibited a negative correlation between age at diagnosis and m.3243A>G heteroplasmy level, with *P*-values of < 0.001 and 0.004, respectively.

**Table 2:** Characteristics between MDM-score ≥ 3 and MDM-score < 3 groups with MDM.

Characteristics	MDM-score ≥ 3	MDM-score < 3	*P* Value
N	23	25	
Female, n (%)	9 (39.1)	12 (48.0)	0.536
Age of diagnosis (year)	29.9 (25.8-32.2)	33.6 (23.9-38.4)	0.667
Anthropometric factors			
Body mass index(kg/m^2^)	19.26 ± 2.45	22.28 ± 4.80	0.009
Height (cm)	161.57 ± 9.76	162.54 ± 9.30	0.723
Weight (kg)	50.60 ± 9.78	59.97 ± 18.03	0.031
Systolic blood pressure (mmHg)	114.00 ± 9.66	120.44 ± 12.73	0.070
Diastolic blood pressure (mmHg)	75.05 ± 8.77	78.76 ± 10.50	0.198
Biochemical data			
HbA1c (%)	10.32 ± 2.85	10.11 ± 2.94	0.801
Fasting plasma glucose (mmol/L)	8.20 (6.60-10.62)	7.50 (6.81-9.70)	0.432
2 h Postprandial plasma glucose (mmol/L)	16.38 ± 6.29	15.21 ± 6.64	0.542
Fasting C-peptid (ng/mL)	0.39 (0.27-0.54)	0.51 (0.31-0.70)	0.237
2 h Postprandial C-peptide (ng/mL)	0.82 (0.56-1.56)	1.17 (0.93-1.81)	0.067
Triglyceride (mmol/L)	1.80 (1.22-2.54)	2.06 (1.36-2.55)	0.322
Total Cholesterol (mmol/L)	4.72 ± 1.29	4.77 ± 1.20	0.886
High density lipoprotein (mmol/L)	1.12 (0.94-1.31)	1.03 (0.97-1.21)	0.136
Low density lipoprotein (mmol/L)	3.01 ± 0.71	2.92 ± 0.96	0.714
Questionnaire data			
Diet treatment, n (%)	9 (39.1)	10 (40.0)	0.951
Physical activity treatment, n (%)	7 (30.4)	8 (32.0)	0.907
Insulin treatment, n (%)	16 (69.6)	1 (4.0)	<0.001
Metformin treatment, n (%)	5 (21.7)	6 (24.0)	0.852
Sulphonyl treatment, n (%)	2 (8.7)	2 (8.0)	1.000
Acarbose treatment, n (%)	5 (21.7)	1 (4.0)	0.156
GLP-1 treatment, n (%)	0 (0.0)	1 (4.0)	1.000
Family history of diabetes, n (%)	17 (73.9)	2 (8.0)	<0.001
Diabetic ketoacidosis history, n (%)	3 (13.0)	4 (16.0)	1.000

Data were presented as number (%) for categorical variables, median (Q1-Q3) or mean ± standard deviation for continuous variables. MDM, Mitochondrial diabetes mellitus; HbA1c, hemoglobin A1c.

### 3.5 Accuracy of MDM-score for screening MDM patients in clinical T2DM

Our clinical T2DM cohort identified 23 MDM cases, yielding an AUC of 0.612 (95% CI: 0.540-0.683, *P <*0.001) in ROC curve analysis. The results showed a sensitivity of 0.479, a specificity of 0.744, a positive predictive value (PPV) of 0.019, and a negative predictive value (NPV) of 0.993, as detailed in **Table 3**. Although our overall performance was somewhat lower than that reported in the developer’s study, the MDM-score approach allowed us to identify nearly half of the MDM cases among diabetic participants.

**Table 3:** Performance of MDM-score for screening MDM patients in different clinical T2DM corhort.

Screening Patients	Total (n)	SEN (%)	SPE (%)	PPV (%)	NPV (%)	AUC (95% CI)	*P* Value
Newly diagnosed outpatients (Our study)	4829	47.9	74.4	1.9	99.3	0.612 (0.540-0.683)	<0.001
Inpatients (Tian et al) (20)	1037	100	69.9	1.6	100	0.884 (0.801-0.967)	0.003

MDM, Mitochondrial diabetes mellitus; T2DM, type 2 diabetes mellitus; SEN, sensitivity; SPE, specificity; PPV, positive predictive value; NPV, negative predictive value; AUC, Area Under Curve.

## 4 Discussion

Mitochondrial diabetes mellitus (MDM) is a rare but significant form of diabetes characterized by maternal inheritance and associated with specific genetic mutations in mitochondrial DNA, particularly the m.3243A>G mutation. This condition presents unique challenges to clinicians due to its overlapping clinical features with more common forms of diabetes, such as T2DM. Our study included outpatients with newly diagnosed diabetes from 46 tertiary hospitals across 24 provinces in China. Genetic testing revealed that the prevalence of MDM in clinical T2DM is 0.97%. This finding is notable as it exceeds the previously reported prevalence of 0.5% in both Chinese and European populations (20, 23), suggesting a higher occurrence of MDM that may have been underestimated in earlier studies. With this multicenter cohort study, we validated for the first time the accuracy of the MDM-score developed by Chinese researchers for screening MDM in early-onset newly diagnosed diabetes, making the first large-scale study on clinical screening for MDM in China.

MDM has several distinct clinical features that differentiate it from other forms of diabetes. These features are typically associated with MDM and are supported by previous studies: (1) maternal inheritance of diabetes and deafness; (2) early middle aged onset of diabetes and deafness; (3) thin and short stature; (4) absence of anti-GAD autoantibodies; (5) low heteroplasmy in leukocytes; (6) high incidence of neurosensory deafness; (7) early requirement for insulin therapy due to the progressive insulin secretory defect (24–30). Tian et al. reviewed the characteristics of the Chinese MDM population and found that 92.6% had a young age at diabetes diagnosis (≤ 45 years), 94% had a BMI <24 kg/m², 85.4% had abnormal hearing, 61.4% received insulin injections, and 98% had a maternal history of hyperglycemia or diabetes (20). They then developed the MDM score tool, which was effectively validated in hospitalized MDM patients. In this study, we found newly diagnosed MDM patients rarely exhibit all these clinical features, and evaluated the performance of the MDM-score as a screening tool for identifying patients with MDM among those newly diagnosed with T2DM. By analyzing a cohort of 4952 newly diagnosed diabetes patients across various hospitals in China, we aimed to delineate the clinical characteristics distinguishing MDM from T2DM and to assess the utility of the MDM-score through receiver operating characteristic (ROC) curve analysis. Our findings demonstrated a modest area under the curve (AUC) of 0.612 (95% CI: 0.540-0.683, *P* < 0.001), suggesting that the MDM-score may help identify nearly half of the patients with MDM. This result is not very optimal compared to previous study, but the participants included in this study were outpatients attending clinics. In general, the onset of MDM is much earlier in outpatients, and the clinical phenotype is atypical. The MDM-score tool will play a significant role in different application scenarios, as it can help clinicians identify MDM patients from the large number of diabetes patients at an earlier stage. Notably, MDM-score’s performance warrants further refinement.

Our study indicated that MDM patients did not differ significantly from T2DM regarding family history of diabetes and insulin therapy. However, MDM patients were diagnosed with diabetes at a younger age and had lower BMI, height, weight and lower fasting C-peptide (*P* < 0.05). These metrics may help to further improve the performance of the MDM scoring system. Furthermore, the phenotypes of m.3243A>G are highly variable, with different symptoms in different patients. when comparing the clinical phenotypes of MDM patients with MDM score ≥ 3 and MDM score < 3, there were some differences in body weight (*P*=0.031). A study on adult patients with mitochondrial disease indicated that height could serve as a clinical biomarker for disease burden in MDM patients (31). Another cohort study involving 136 patients with m.3243A>G MDM identified several common phenotypes, including family histories (84.51%), hearing loss (85.71%), underweight (41.58%) (32). As mtDNA mutations lead to impaired mitochondrial ATP synthesis, this may affect the mechanisms of reduced muscle mass and exercise intolerance in childhood with early-onset mitochondrial disease, which in return increases apoptosis and cell death in the growth plate, ultimately leading to poor growth and short stature (33). On the other hand, patients with m.3243A>G suffer from malnutrition and weight loss due to common gastrointestinal symptoms, including reduced intake from poor appetite and delayed gastric emptying (34). This observation indicates that height and weight in MDM are likely lower than in other diabetes subtypes. These factors may serve as distinguishing indicators, in contrast to the BMI, which may not adequately reflect individual growth patterns.

The clinical phenotype of MDM can manifest as either type 1 or type 2 diabetes, depending on the severity of insulin deficiency. It is often misdiagnosed as T2DM, particularly when detectable fasting C-peptide levels are present in the early stages of MDM, which shares clinical features with T2DM. Several studies have shown that patients with MDM do not have GADA, unlike classical T1DM (35, 36). We included a study population of patients with newly diagnosed diabetes, all of whom tested negative for GADA, thereby excluding most classical autoimmune T1DM cases. We observed 14 clinical T1DM patients (fasting C-peptide < 0.01 ng/ml) with an MDM-score of ≥ 3. However, genetic testing confirmed that none of them had MDM. This finding further indicates that the MDM-score is not suitable for screening MDM in clinical T1DM patients.

The present study demonstrates the utility of the MDM-score in screening for MDM among patients diagnosed with T2DM. This is a significant advancement in our understanding of how to identify patients at risk for MDM, particularly given the known association of the m.3243A>G heteroplasmy level with mitochondrial dysfunction and its implications for metabolic health. Elevated m.3243A>G heteroplasmy was associated with reduced strength, cognitive, metabolic, and cardiovascular functioning, and increased risk of stroke mortality (18, 37, 38). Similar to previous findings, our study showed a negative correlation between age at diagnosis of diabetes and the m.3243A>G heteroplasmy level (*P* < 0.001; *P* = 0.004). We found that heteroplasmy was higher in the MDM score ≥ 3 group than in the MDM score < 3 group (*P* = 0.0281), suggesting that the MDM score may reflect the severity and progression of the disease. As the level of heterogeneity in leukocytes decreases with age, the MDM-score serves as a rough screen at the first gate, which can be further combined with the level of heterogeneity after patients have refined their genetic testing to determine the overall prognosis of the disease.

Our multicenter cohort indicated that the MDM-score effectively screens for MDM cases in newly diagnosed diabetes patients, facilitating early and precise management. We also recommend early insulin therapy for managing MDM. Many agents are known to be detrimental to mitochondrial function, including some antibiotics, anti-epileptic drugs, nucleoside analogue reverse transcriptase inhibitors and metformin (5, 39, 40). Statins are prohibited due to their potential to increase the risk of myopathy in patients with MDM (41). The appropriate use of mitochondrial function-enhancing drugs, such as coenzyme Q10 and thiamine, may benefit patients with MDM (42–44). Regular audiologic monitoring is essential for early intervention. Additionally, long-term follow-up and genetic counseling for relatives are necessary, along with prompt attention to cases of renal failure or cardiac problems.

There are some limitations. First, the study population was exclusively Chinese, so the validity of the MDM-score should be assessed in diverse ethnic and geographic groups, such as those in Europe and America. Second, all participants did not receive a standardized hearing-related physical examination or electronic testing. In the developer’s study, 1 in 5 patients with MDM had hearing impairment (20%) during follow-up, which means that our study may have missed patients with early sensorineural hearing impairment (45). Although early hearing impairment is typically mild, it may affect the sensitivity and positive predictive value (PPV) of the MDM score when a cut-off of ≥ 3 is used for screening.

In conclusion, MDM is a complex and rare disease that needs a thorough method for diagnosis and management. The MDM-score is not widely recognized or used globally. Our study is the first to show that the MDM-score can be applied to newly diagnosed diabetes patients. This suggests that for newly diagnosed diabetes patients, the MDM-score, along with the detection of GADA, is helpful in distinguishing MDM from T2DM and T1DM. This guidance assist clinicians in selecting suitable patients for further genetic testing, which helps reduce the risk of misdiagnosis in early-stage MDM patients. Some metrics such as height, weight, and fasting C-peptide levels may help to further improve the performance of the MDM scoring system in the future.

## Data Availability

The original contributions presented in the study are included in the article/**Supplementary Material**. Further inquiries can be directed to the corresponding authors.
