# Supplementary material for: Evaluation of the MDM-score system for screening mitochondrial diabetes mellitus in newly diagnosed diabetes patients: a multi-center cohort study in China
Source: Front Endocrinol (Lausanne). 2024 Dec 19;15:1511101. doi: 10.3389/fendo.2024.1511101 (PMC11693586; doi:10.3389/fendo.2024.1511101)
Supplement: Supplementary file 1 [file DataSheet1.docx]

**Supplementary Materials**

**TABLE S1** Subjects to targeted sequencing of 37 genes in this study

| Chromosome location | Gene name | Chromosome location | Gene name |
| --- | --- | --- | --- |
| Chr1 | LMNA | Chr9 | CEL |
| Chr1 | PPP1R15B | Chr10 | PCBD1 |
| Chr2 | DCAF17 | Chr10 | SLC29A3 |
| Chr2 | NEUROD1 | Chr11 | INS |
| Chr2 | KLF11 | Chr11 | KCNJ11 |
| Chr3 | PPARG | Chr11 | ABCC8 |
| Chr3 | APPL1 | Chr11 | PAX6 |
| Chr4 | WFS1 | Chr12 | HNF1A |
| Chr4 | CISD2 | Chr13 | PDX1 |
| Chr4 | TRMT10A | Chr13 | DNAJC3 |
| Chr5 | PIK3R1 | Chr15 | PLIN1 |
| Chr6 | ZFP57 | Chr17 | HNF1B |
| Chr6 | RFX6 | Chr18 | GATA6 |
| Chr6 | ZBTB2 | Chr19 | INSR |
| Chr7 | GCK | Chr19 | DYRK1B |
| Chr7 | PAX4 | Chr19 | AKT2 |
| Chr8 | BLK | Chr19 | PDLD1 |
| Chr8 | GATA4 | Chr20 | HNF4A |
| ChrM | MTTL1 |  |  |


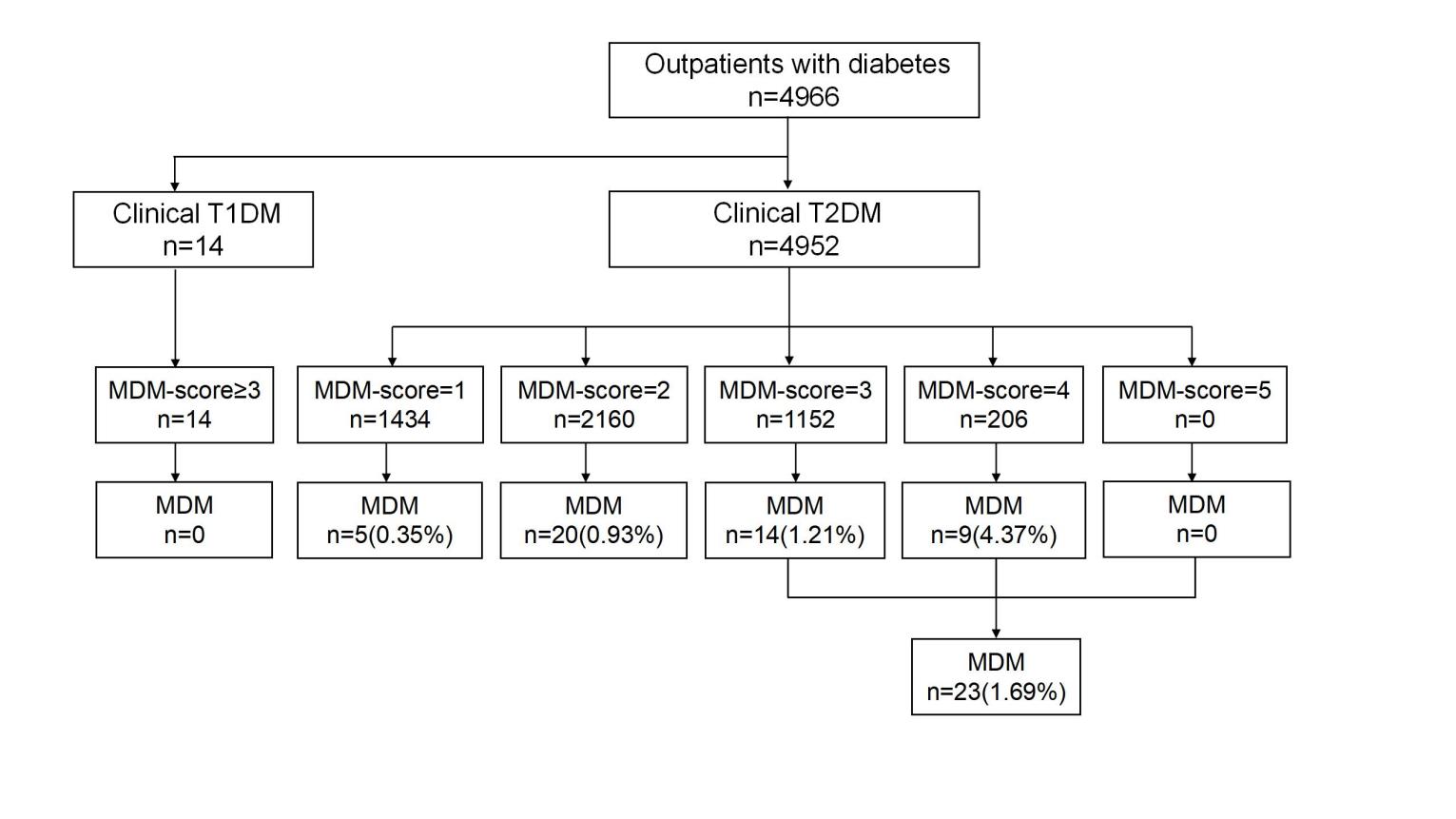


**FIGURE S1** Application of the MDM‑score in clinical screening for MDM patients.

MDM-score: A summed score of the following parameters: (1) Age at diagnosis of diabetes ≤ 45 years old (1 point); (2) family history of diabetes and/or abnormal hearing on their mother’s side (1 point); (3) BMI < 24.0 kg/m^2^ (1 point); (4) abnormal hearing (1 point); (5) insulin treatment (1 point). MDM, mitochondrial diabetes mellitus; T1DM, type 1 diabetes mellitus; T2DM, type 2 diabetes mellitus.
